# Immunophenotyping of septic shock patients with endotheliopathy: focus on monocyte subtypes and Immune regulatory molecules

**DOI:** 10.3389/fimmu.2025.1656815

**Published:** 2025-12-04

**Authors:** Saif Al-Haidar, Martin Schønemann-Lund, Mikkel Gybel-Brask, Jakob Stensballe, Lars Andresen, Pär I. Johansson, Morten H. Bestle, Søren Skov

**Affiliations:** 1 Laboratory of Immunology, Section for Biomedicine, Faculty of Health and Medical Sciences, University of Copenhagen, Copenhagen, Denmark; 2 Department of Anesthesia and Intensive Care, Copenhagen University Hospital - North Zealand, Hilleroed, Denmark; 3 Capital Region Blood Bank, Sect. for Transfusion Medicine, Department of Clinical Immunology, Copenhagen University Hospital - Rigshospitalet, Copenhagen, Denmark; 4 Department of Clinical Medicine, University of Copenhagen, Copenhagen, Denmark; 5 Department of Anesthesia, Surgery and Trauma Center, Copenhagen University Hospital - Rigshospitalet, Copenhagen, Denmark

**Keywords:** septic shock, monocytes, immune checkpoint molecules, immunophenotyping, endothelium, cytokine storm, flow cytometry, immune dysfunction

## Abstract

**Background:**

Septic shock is characterized by dysregulation of the host response to infection which results in life-threatening organ dysfunction that can be partly attributed to immune alterations and endothelial dysfunction (endotheliopathy). This lethal condition is dynamic, complex, and heterogeneous.

**Aim:**

Thus, an exploratory broad phenotypic and functional analysis of circulating immune cells and mediators were carried out to better understand the role of the immune system in a subgroup of septic shock patients with endotheliopathy defined by increased levels of soluble thrombomodulin (sTM). In this regard, especially the immune status of monocyte subtypes (classical, intermediate, and nonclassical) was investigated for surface thrombomodulin (TM), MHC class II molecules (HLADR, -DQ, and -DP) and immunomodulatory surface receptors (TREM-1, CD137, VISTA, HVEM and BTLA).

**Result and conclusion:**

Our comprehensive immunophenotypic analysis on a septic shock cohort with endotheliopathy identified distinct immune perturbation patterns that potentially can lead to novel treatment avenues in the management of this life-threatening condition.

## 1 Introduction

A major pathogenesis of septic shock is a dysregulated host response to infection (1), potentially leading to failure of several organs, including the vascular endothelium (endotheliopathy) and the immune system (immune dysregulation) (2–4). Previous evidence has demonstrated that the hallmark of immune dysregulation is monocytic immunoparalysis (5–7). Monocytes are a heterogeneous cell population that can be divided into three subpopulations in humans by their relative expression of CD14 (LPS receptor) and CD16 (Fc gamma receptor IIIa). In recent years, our understanding of the roles of each monocyte subtype (CD14^++^/CD16^−^ classical, CD14^++^/CD16^+^ intermediate, and CD14^+^/CD16^++^ nonclassical) has improved. Under inflammatory conditions, classical monocytes infiltrate organs more frequently than intermediate- and nonclassical monocytes (8). In tissues, classical monocytes perform phagocytosis, produce inflammatory mediators and differentiate to monocyte-derived cells. Intermediate monocytes are believed to be responsible for the proliferation and stimulation of T cells based on their increased levels of surface molecules involved in antigen presentation and T cell interaction (9). The more mature and longer-lived nonclassical monocyte is thought to survey the luminal side of vascular endothelium (10). The patrolling features of nonclassical monocytes allow them to monitor endothelial cell integrity, scavenge microparticles and remove cellular debris from the endothelial surface in case of cell death (11, 12). In sepsis, CD16^+^ (intermediate and/or nonclassical) monocytes expand (13). Furthermore, monocytes in general acquire features of immunoparalysis in septic patients. Such monocytic immunoparalysis is evident by a markedly reduced major histocompatibility complex class II (MHC II) expression, reduced co-stimulatory molecules (CD80/86), and a profound reduction of their ability to produce LPS-induced inflammatory cytokines such as TNF-α and IFN-γ *in vitro (*14, 15). MHC class II molecules are responsible for antigen presentation to T cells and between several MHC II isotypes (HLA-DR, -DQ, and -DP) monocytic human leukocyte antigen-DR (HLA-DR) is considered the gold standard for the identification of immunoparalysis-induced immunosuppression (16). Moreover, the diminished expression of monocytic HLA-DR correlates with the risk of secondary infections and mortality in septic shock patients (17).

In septic shock, the dysregulated host response may lead to endothelial activation and subsequent dysfunction, termed endotheliopathy. Here, endothelial integrity is compromised (e.g. endothelial cell damage and loss of glycocalyx) and coagulation disorders such as disseminated intravascular coagulation (DIC) are more frequent (18). Biomarkers of endothelial injury such as soluble thrombomodulin (sTM) are elevated in patients with septic shock (19). Furthermore, a positive correlation between sTM and organ failure and mortality has been found in septic patients (20, 21). sTM comes from the extracellular portion of the transmembrane thrombomodulin (TM) receptor, which predominantly is expressed on the surface of vascular endothelium. Endothelial TM functions as an anticoagulant factor by e.g., activating protein C. In addition, TM has also been detected on human monocytes but the contribution and function of monocytic TM in endotheliopathy and sepsis is unknown (22).

Re-balancing the immune dysregulation in sepsis and septic shock may lead to better outcomes for patients. In line with this, immunomodulatory strategies that inhibit the detrimental immune response or boost the immune system in the immunosuppressed state have recently become a subject of basic and translational research in the field of sepsis (23). Based on the expression of immune checkpoint molecules such as PD-1 and TIM-3, T cells in septic patients seem to be more activated and may exhibit a trend toward exhaustion (24, 25). Nevertheless, the role of other immunomodulating molecules such as CD137, VISTA, HVEM and BTLA is currently scarce in sepsis-mediated immune dysregulation. CD137 (also known as 4-1BB), is a co-stimulatory receptor preferentially found on activated T-cells (particularly CD8^+^ T cells), NK-cells, neutrophils, and monocytes (26). On the other hand, VISTA (also known as DD1a, Dies1, Gi24, PD-1H) have the highest level of expression on myeloid cells including monocytes although the role of this receptor on monocytes is still controversial (27). The receptor HVEM functions as molecular switch, because it has both immune suppressive (BTLA) and stimulating (CD160, LIGHT and lymphotoxin-α) ligands (28). TREM-1 can be described as an endogenous mediator of the inflammatory response, which can amplify the host immune response. Regarding sepsis, there is consensus for monocytes from septic patients upregulate TREM-1 (29). However, there are conflicting results of the expression pattern of TREM-1 on neutrophils (30).

In this study, an exploratory broad phenotypic analysis of circulating immune cells and mediators (cytokines) were carried out to better understand the role of the immune system in a subgroup of septic shock patients with endotheliopathy defined by sTM. In this regard, especially the immune status of monocyte subtypes was investigated using TM, HLA-DR, -DQ, and -DP and five immunomodulating (TREM-1, CD137, VISTA, HVEM and BTLA) surface receptors.

## 2 Materials and methods

### 2.1 Study design and enrollment

This study was conducted as part of the COMBAT-SHINE randomized controlled trial (31). The COMBAT-SHINE study protocol is described in detail elsewhere (32). In short, patients aged 18 years or older were considered eligible when they complied with the 2016 International Consensus Definitions of septic shock (1), were admitted to an intensive care unit (ICU), and had sTM >10 ng/mL within 12 hours of septic shock diagnosis. For the present study, only patients from Copenhagen University Hospital – North Zealand (CUH) were included, as regulatory approval for sample access was exclusively granted for this site. Recruitment was between 28^th^ September 2021 and 28^th^ February 2022. During the same period, healthy age-matched volunteers with no significant concomitant acute or chronic illnesses were also enrolled. Patient clinical data extracted from the electronic medical record were retrospectively reviewed, while age and sex were recorded for each healthy control.

### 2.2 Soluble thrombomodulin measurements (ELISA)

The sTM-based stratification of patients was measured by using a lateral flow immunoassay (BioPorto Diagnostics A/S) when screening septic shock patients for eligibility for inclusion in the COMBAT-SHINE trial. EDTA-plasma was separated after centrifuging at 3000 rounds per minute for 10 min directly after collection and used for the analysis.

### 2.3 Blood sample collection and serum isolation

~15 mL blood was collected using BD Vacutainer^®^ Safety-Lok™ Blood Collection Set system (BD, Cat. # 368654) and sodium heparin tubes (BD, Cat. # 367880), at baseline (before administration of the investigational drug, Iloprost). The blood samples were kept at room temperature and analyzed within 12 hours.

Serum was prepared by transferring 0.5 mL of blood into 5 ml Eppendorf tube, and the blood was allowed to clot undisturbed at room temperature. Clot was then removed by centrifuging at 1000 ×g for 5 minutes in a refrigerated centrifuge (10 °C). The separated sera were transferred into cryogenic storage vials and stored at −80 °C until further analysis.

### 2.4 Flow cytometry analysis

Briefly, whole blood was lysed by 1x red blood cell (RBC) lysis buffer (eBioscience™, Cat. # 00-4333-57) and washed two-three times with phosphate-buffered saline (PBS) according to manufacturer’s instructions. The samples were transferred to 5 ml FACS tubes and subsequently resuspended in 50 μL fragment crystallizable receptors (FcRs) block reagent (Miltenyi Biotech, Cat. # 553142) diluted 1:100 in FACS buffer (PBS + 2% fetal bovine serum) and incubated on ice for 10 minutes. After FcR-blocking, cells were washed in 1 mL FACS buffer prior to staining. Samples were surface stained for 15 minutes with fluorochrome-conjugated surface antibodies as listed in **Supplementary Tables 1**, **2**. All antibodies were titrated prior to the study. Tandem signal enhancer solution (Miltenyi Biotech, Cat. #, 130-099-887) and 7-AAD viability dye (Miltenyi Biotech, Cat. # 130-111-568) was included in the MasterMix. After washing, the samples were analyzed on a MACSQuant Analyzer 16 flow cytometer. All downstream analyses were performed in FlowJo version 10.8.1 software (Tree Star Inc.). For gating strategies see **Supplementary Figures 1, 2**. For proper gating, fluorescence minus one (FMO) control were used. Unbiased hierarchical cluster profile of all markers used in this study can be found in **Supplementary Figures 3**.

### 2.5 Cytokine measurements (Meso Scale)

Serum was thawed at room temperatures and assayed for the presence of IFN-γ, IL-1β, IL-4, IL-6, IL-8, IL-10 and TNF-α using the multiplex cytokine assay (V-PLEX Viral Panel 2 Human Kit, Meso Scale Diagnostics, MSD, Cat. # K15346D) following the recommendations of the manufacturer. MSD plates were analyzed on MESO QuickPlex SQ 120 (AI0AA-0, MSD) instrument using the MSD discovery workbench, version 4.0.13 software (Meso Scale Diagnostics). All standards and samples were measured in duplicate.

### 2.6 Statistical and computational analyses

Statistics and graphical representations were performed using R version 4.1.2 with RStudio integrated development environment (IDE, https://www.rstudio.com/products/rstudio/).The built-in R functions prcomp() and pheatmap (version 1.0.12) and ggplot (version 2 3.3.5) packages were used to perform a Principal Component Analysis (PCA), heatmap and visualization analyses. PCA and heatmap were conducted on standardized (rescaled) data. Furthermore, heatmap was based on hierarchical clustering by Euclidean distance.

For cytokine expression levels, statistical comparison between healthy and patients was done using a Welch’s t test (significant difference is reported with the actual p-value). For flow cytometry data, statistical significance was assessed using two-tailed Mann-Whitney U-test to compare each cell type from patients with the same cell type from healthy controls.

For all experiments, the level of statistical significance was determined by a p value of <0.05 and presented as follows: ns: p > 0.05, *: p ≤ 0.05, **: p ≤ 0.01, ***: p ≤ 0.001, ****: p ≤ 0.0001.

## 3 Results

### 3.1 Clinical characterization of patient cohort and quantification of cytokine levels

Patients with septic shock and sTM above 10 ng/mL were included in the study. The clinical characteristics of healthy volunteers and patients are presented in **Table 1**. Due to the minor differences observed after stratification by sex (**Supplementary Figures 8, 9**), the healthy male and healthy females were considered as one group.

**Table 1:** Patient characteristics and clinical features.

Clinical Parameters	Healthy volunteers (n = 10)	Septic shock patients (n = 7)
Age, median [range]	48 [35-58]	55 [33-72]
Male sex, number (%)	5 (50%)	7 (100%)
sTM in ng/mL, mean [range]	NA	23.9 [12.9-31.6]
SOFA score, mean	NA	11.1
SOFA score respiratory	NA	3.1
SOFA score cardiovascular	NA	3.9
SOFA score hematology	NA	0.3
SOFA score liver	NA	1.0
SOFA score renal	NA	2.9
Highest creatinine level at baseline, mean [range] - μmol/L	NA	286.7 [73-519]
Highest bilirubin level at baseline, mean [range] - μmol/L	NA	35 [5-71]
Primary infectious focus		
Gastrointestinal tract	NA	4 (57%)
Lungs	NA	2 (29%)
Urinary tract	NA	0 (0%)
Skin or soft tissue	NA	1 (14%)
Other	NA	0 (0%)
Unknown	NA	0 (0%)
Time from sepsis diagnosis to blood sampling, mean [range] – hours	NA	3.8 [0.5-11.8]

NA, Not applicable; SOFA, Sequential Organ Failure Assessment.

**Figure 1:**
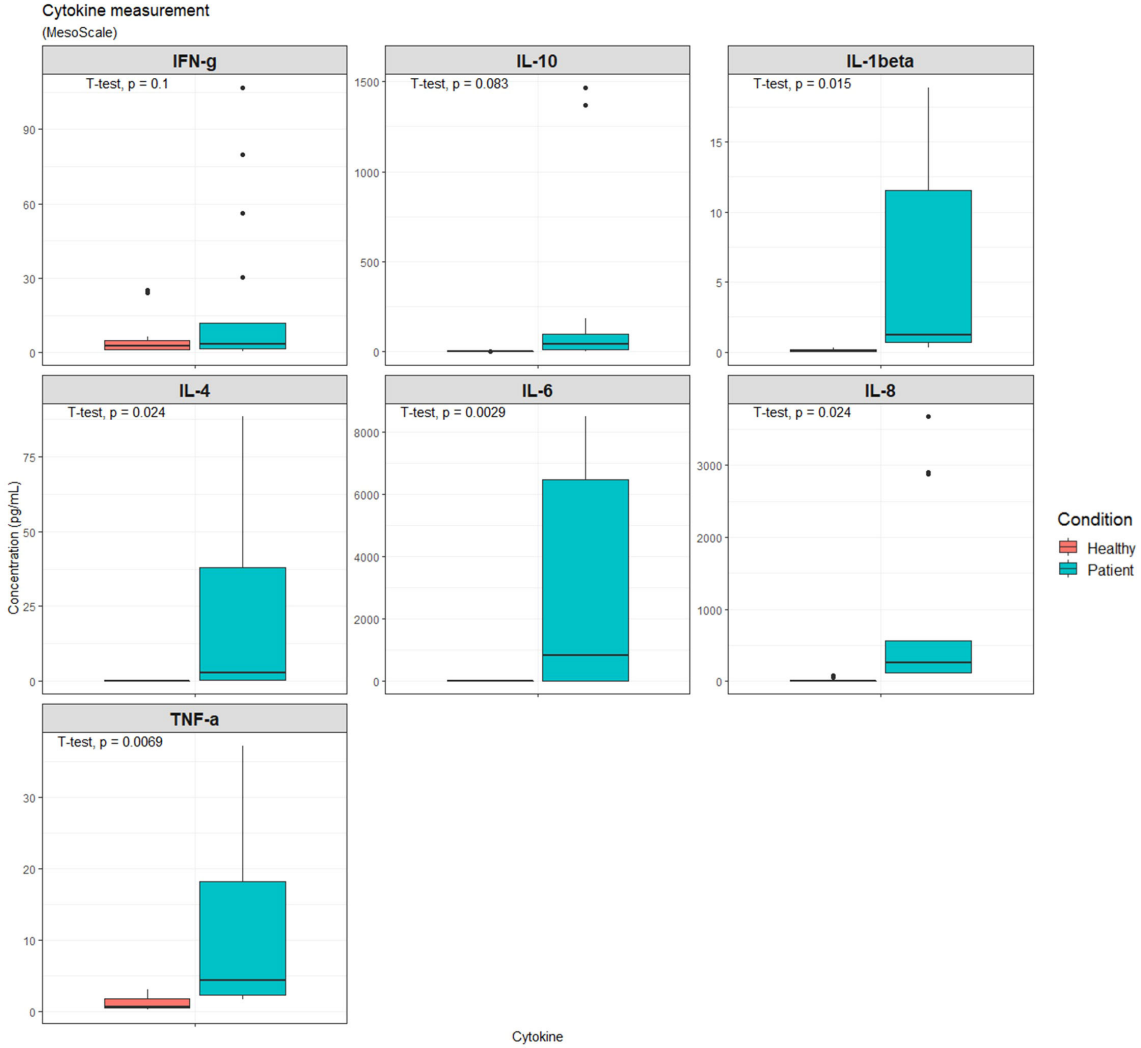
Expression of indicated cytokines in the serum from septic shock patients (n=7) or healthy volunteers (n=10). Cytokine expression levels using Meso Scale. IFN-γ: Interferon gamma, IL-10: Interleukin-10, IL-1β: Interleukin-1 beta, IL-4: Interleukin-4, IL-6: Interleukin-6, IL-8: Interleukin-8, TNF-α: Tumor necrosis factor alpha.

To define the immune response of the patient cohort we quantified the cytokines commonly associated with infections. Compared with healthy volunteers, both pro-inflammatory (IFN-γ, IL-1β, IL-6, IL-8, and TNF-α) and anti-inflammatory (IL-4 and IL-10) cytokines were elevated in patients with septic shock (**Figure 1**). However, the increase in IL-10 and IFN-γ were not statistically significant.

### 3.2 The composition of immune cells in septic shock patients with endotheliopathy

**Figure 2:**
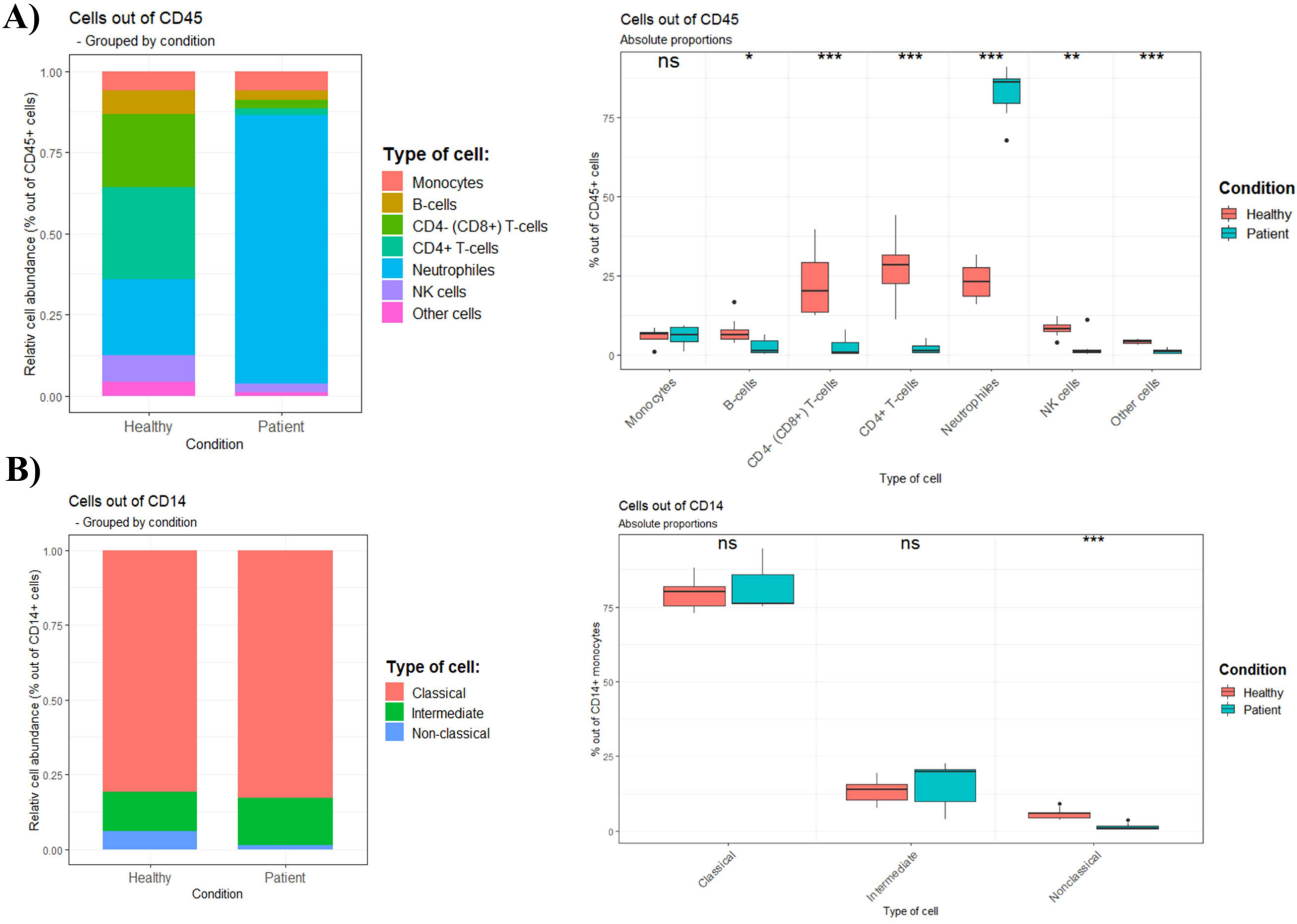
Relative and absolute proportions of **(A)** six immune cell subsets and of **(B)** monocyte subtypes in healthy controls (n=10) and septic shock patients (n=7). Proportions are defined as frequency of immune cells relatively to total CD45^+^ cells for **(A)**, while relatively to total CD14^+^ monocytes for **(B)**.

Relative and absolute proportions of six immune cell subsets and of monocyte subtypes are shown in **Figure 2**. Based on cellular immunophenotyping, neutrophils are the most prevalent cell type in septic shock patients. In patients a lower proportion of B-cells, CD4^+^ T-cells, CD8^+^ T-cells and NK-cells were also discovered, when compared to the healthy control group. These differences in immune populations were seen despite no change in the total pool of CD45^+^ cells (**Supplementary Figure 7**). Furthermore, no differences in proportion of CD14^+^ monocytes were found between the groups within the CD45^+^ whole blood cell fraction. However, the composition of circulating monocyte subtypes was changed. Here, the distribution of monocyte subtypes revealed a significant reduction in the proportion of nonclassical monocytes (out of CD14^+^ monocytes) in the septic shock cohort.

### 3.3 Immunoregulation in septic shock by costimulatory molecules and immune checkpoints

We further hypothesized that this state of “emergency” might generate immune cells with immunophenotypic abnormalities. Thus, to discover phenotypical differences and demonstrate the status of immune cells in a subgroup of septic shock patients, immunomodulating antigens were evaluated by flow cytometry.

**Figure 3:**
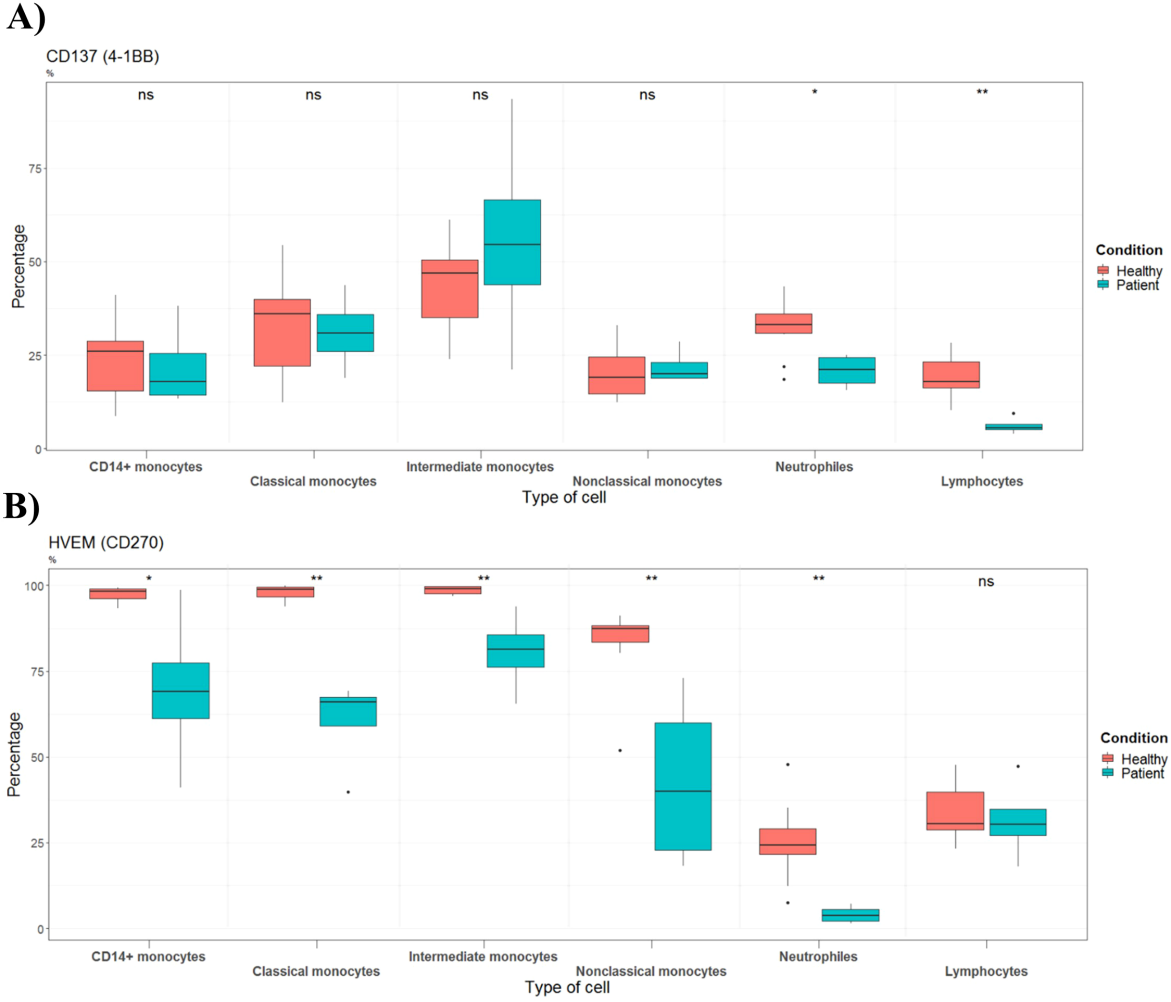
Percentages of cells expressing **(A)** CD137 or **(B)** HVEM/CD270 in healthy controls (n=10) and septic shock patients (n=4). Data are presented as percentage of unique immune cells positive for **(A)** CD137 or HVEM/CD270 as determined by CD137 or HVEM/CD270 FMO control.

**Figure 4:**
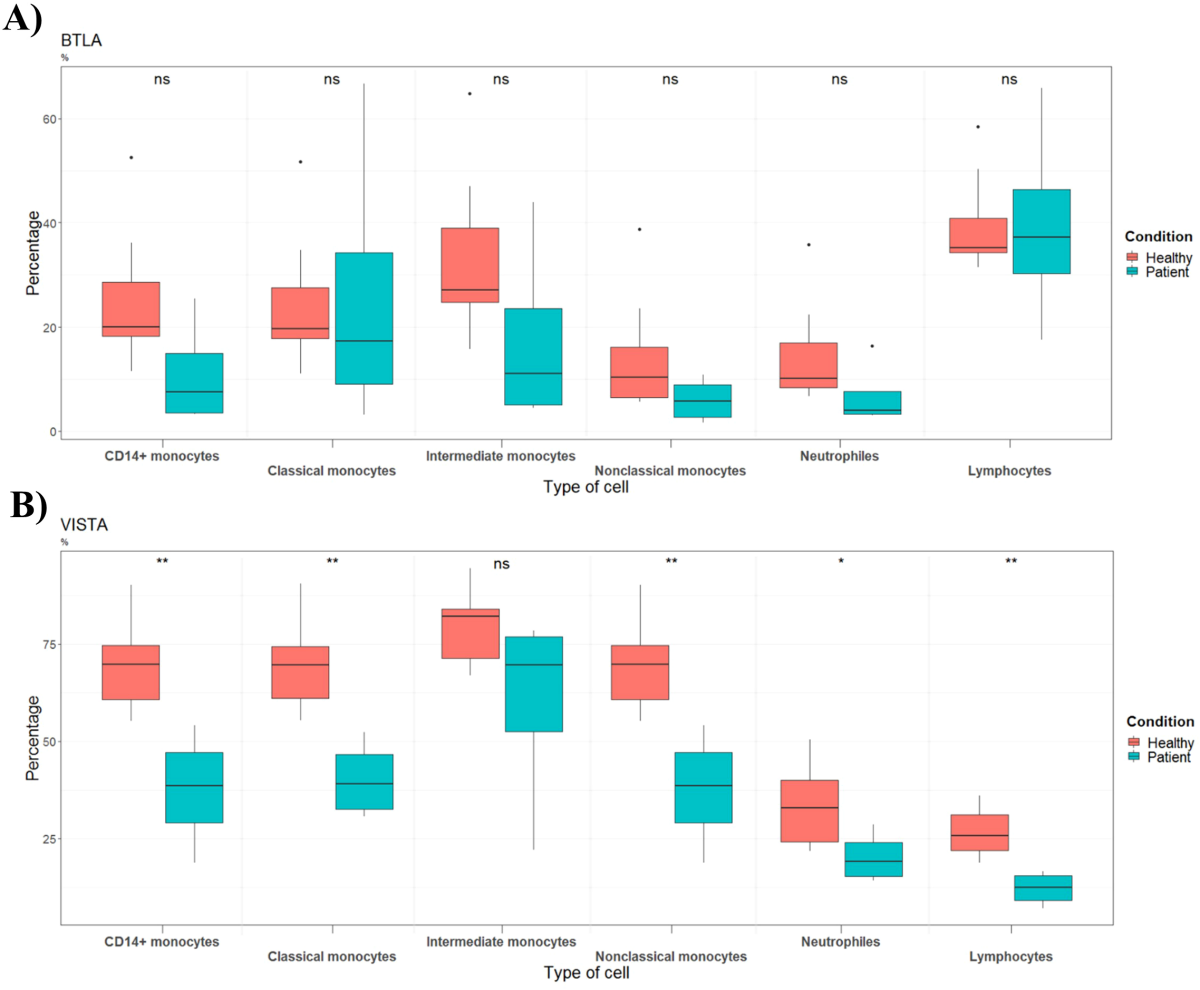
Percentages of cells expressing **(A)** BTLA or **(B)** VISTA in healthy controls (n=10) and septic shock patients (n=4). Data are presented as percentage of unique immune cells positive for A) BTLA or VISTA as determined by BTLA or VISTA FMO control.

The percentage of the costimulatory molecule CD137 on lymphocytes and neutrophils was decreased in patients with septic shock (**Figure 3A**). In addition, among adult septic shock patients, the percentage of HVEM (CD270) was significantly reduced in neutrophils, classical monocytes, intermediate monocytes and nonclassical monocytes (**Figure 3B**). Percentage of BTLA expressing immune cells did not change between groups (**Figure 4A**), indicating that only HVEM is disrupted in the BTLA/HVEM axis.

The newly identified VISTA protein was reduced on lymphocytes, neutrophils, classical and nonclassical monocytes in the patient group as compared with healthy volunteers (**Figure 4B**).

### 3.4 Thrombomodulin, TREM-1 and MHC class II molecules participated in disease progression

**Figure 5:**
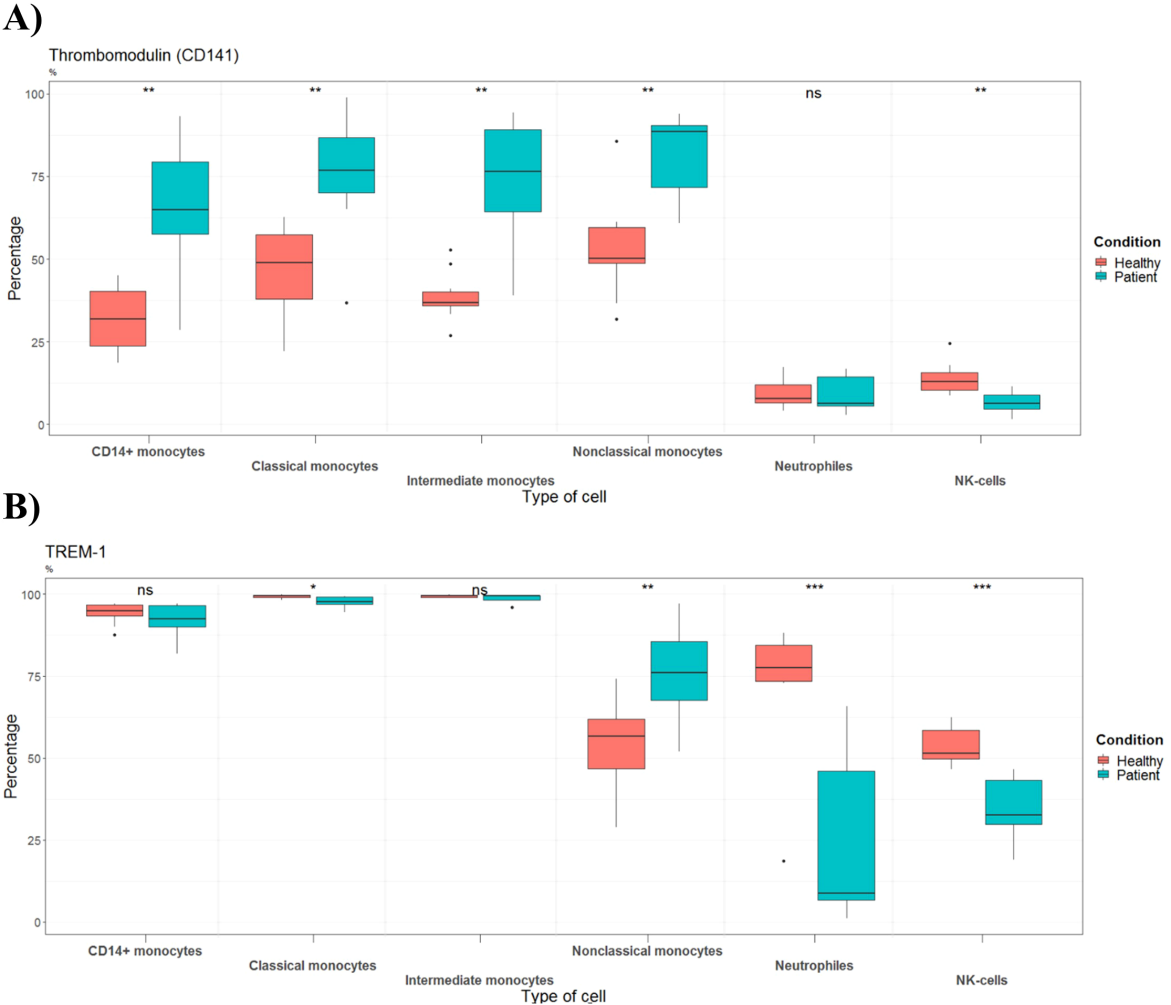
Percentages of cells expressing **(A)** thrombomodulin or **(B)** TREM-1 in healthy controls (n=10) and septic shock patients (n=7). Data are presented as percentage of unique immune cells positive for **(A)** thrombomodulin or TREM-1 as determined by thrombomodulin or TREM-1 FMO control.

The percentage of TM was significantly elevated on all monocyte subtypes from patients when compared to the healthy control group (**Figure 5A**). Futhermore, although a small number of NK-cells have TM, the percentage of these cells expressing TM was significantly reduced in patients. Knowing that, we attempted to test whether immune cells contributed to the elevated sTM in patients at admission. *Ex vivo* incubation of whole blood for 18 hrs, showed that sTM was below the limit of detection in both healthy volunteers and patients, indicating that immune cells did not contribute to the elevation of sTM in patients (**Supplementary Table 3**). To further evaluate the involvement of monocytes in coagulation and inflammation, TREM-1 was measured. Flow cytometric data revealed that septic shock patients had significantly lower percentages of TREM-1 expressing classical monocytes, neutrophils and NK-cells, and increased TREM-1 positive nonclassical monocytes (**Figure 5B**).

**Figure 6:**
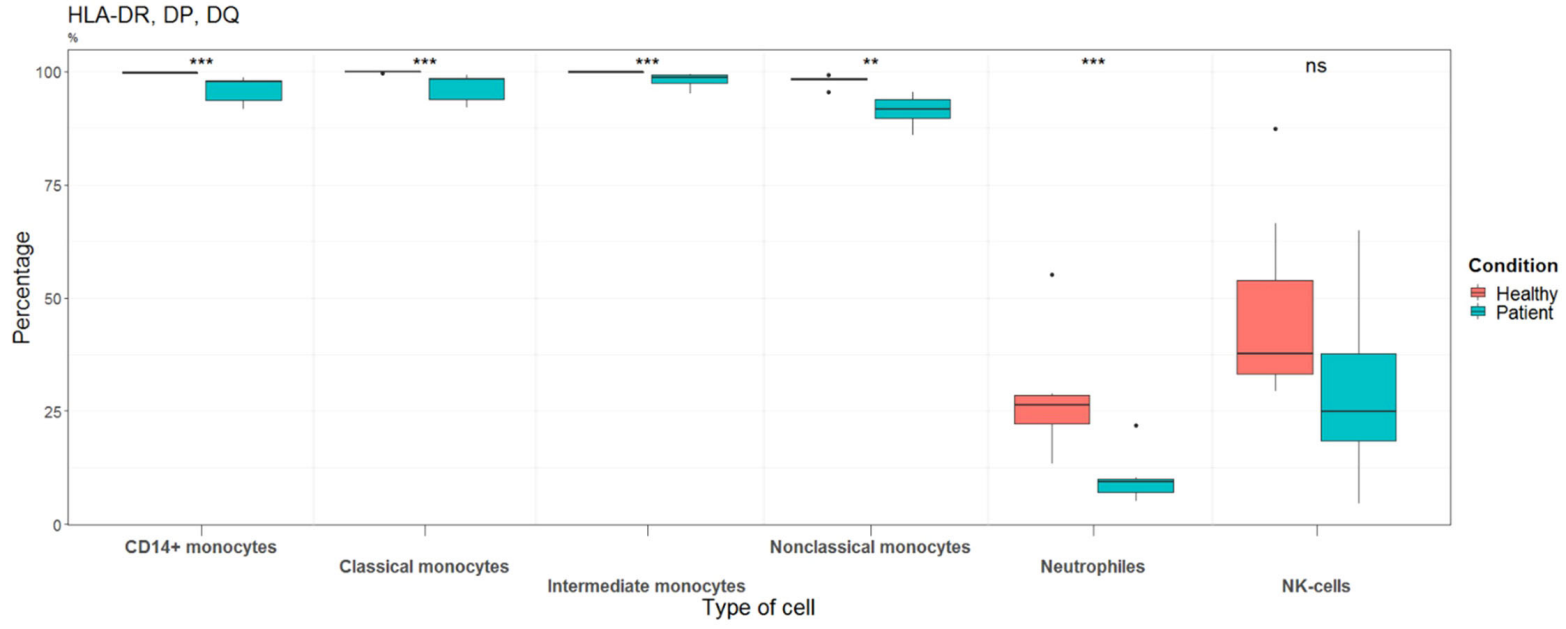
Percentages of cells expressing HLA-DR, DP, DQ in healthy controls (n=10) and septic shock patients (n=7). Data are presented as percentage of unique immune cells positive for HLA-DR, DP, DQ as determined by HLA-DR, DP, DQ FMO control.

To gather additional empirical support, we also included MHC II isotypes in our analysis. As seen in **Figure 6**, the percentage of positive HLA-DR, -DQ, and -DP was significantly decreased in all monocyte subtypes and neutrophils from patients.

### 3.5 Relationships between the monocyte subtypes

**Figure 7:**
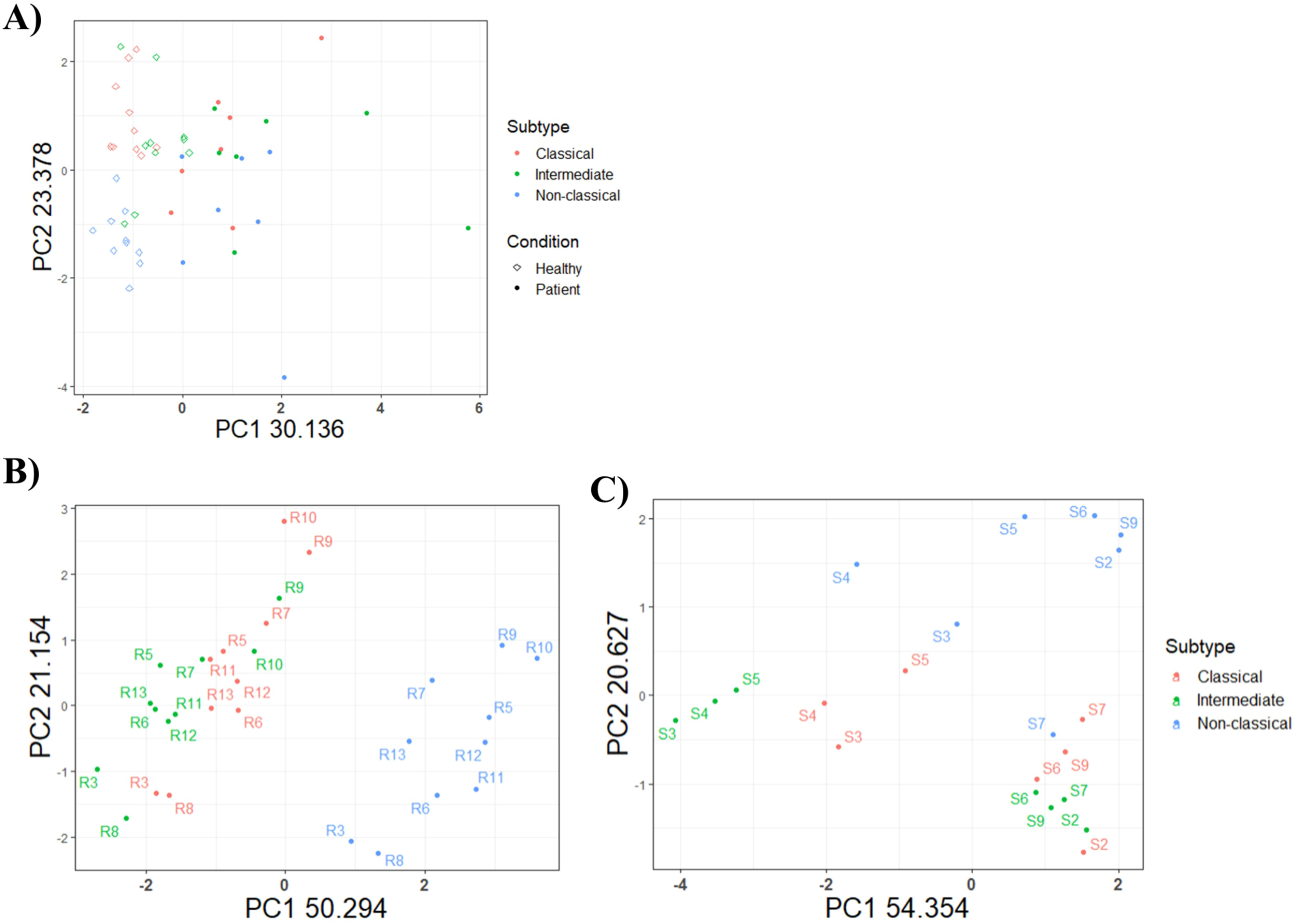
Principal component analysis of monocyte subtypes in **(A)** both groups, **(B)** healthy controls (n=10) and **(C)** septic shock patients (n=7). PCA was based on percentage of positive markers on each unique immune cell. The seven markers used was TREM-1, TM, CD137, CD270, BTLA, VISTA and HLA-DR, DP, DQ. Percentages on the x- and y axes indicate the percentage of variance in the dataset explained by each PC.

Unsupervised principal component analysis (PCA) was used to visualize the relationship among the three subtypes using percentages of the seven markers described thus far (**Figure 7**). PCA revealed two small distinct subgroups of the monocyte subtypes between conditions (**Figure 7A**). Furthermore, the relationships between the three-monocyte subtypes were investigated in both healthy and septic shock patients (**Figures 7B, C**). Here, classical (red), intermediate (green), and nonclassical (blue) monocytes occupied non-overlapping spaces indicating that the subtypes are primarily distinct and have unique protein expression profiles. Nonetheless, there is still some disagreement on the relatedness of the monocyte subtype in the literature (13). However, the PCA showed that the classical (red) and intermediate (green) monocytes were most closely related among the subtypes (**Figures 7B, C**).

## 4 Discussion

In this study, multiparameter evaluations of different cell types have been assessed to explore the immunopathogenesis in patients with septic shock and endotheliopathy. We found that sepsis is characterized by a dysfunctional host immune response affecting all types of immune cells.

### 4.1 Immune perturbation in septic shock patients

To better understand the immune response following septic shock, cellular immunophenotyping of immune cells from both myeloid and lymphoid lineage were conducted. Among the examined cells, neutrophils were the most abundant leukocyte in the systemic circulation of the patients. Noteworthy, the relative increase of neutrophils (neutrophilia), seems to occur at the expense of all lymphocyte subsets (T-cells, B-cells and NK-cells) and both major T-cell subtypes (CD4^+^ and CD8^+^ T-cells). Neutrophilia together with lymphopenia is recognized as an important part of the pathology of sepsis, but the mechanisms responsible for this are not totally understood. Nonetheless, a sustained decreased number of circulating lymphocytes (lymphopenia), have previously been linked to long-term mortality and increased risk of secondary/nosocomial infections (33). Regarding the monocyte numbers in septic patients, there have been discrepancies among studies. Some authors reported an increase in monocytes (monocytosis), while others report decrease (monocytopenia) or no differences (34, 35). Methodological differences, such as the use of whole blood or Ficoll enriched fractions, could account for some of these differences. In this study, no differences in the percentage of CD14^+^ monocytes were found between groups within the CD45^+^ whole blood cell fraction. Intriguingly, because neutrophils made up a much greater proportion of the CD45^+^ leukocyte population, the unchanged prevalence of monocytes compared to other immune cells in septic shock patients could reflect an absolute increase in blood monocyte counts (monocytosis).

Alterations to the circulating monocyte subset distribution occur commonly in various diseases. Comparisons between studies are hampered by employment of different methods and monocytes have been shown to be influenced by PBMC or monocyte purification procedures (36–38). Therefore, we used a fresh whole blood lysis and staining protocol. Here, nonclassical monocytes were found to be decreased in the study cohort. But there is always the question of whether the decrease of nonclassical monocytes represents a net decrease in the peripheral or a compartment shift from blood to tissue. Given the role of nonclassical monocytes in maintaining vascular endothelium, it is not surprising that endotheliopathy in patients will affect the number of these cells. It is tempting to speculate that activation and subsequent dysregulation of the endothelium attract nonclassical monocytes for removing dying endothelial cells. Evidence that supports this is that blockade of the integrin LFA-1 (αLβ2) results in immediate detachment of nonclassical monocytes from endothelium followed by an increase of circulating nonclassical monocytes (39), suggesting that mobilization between a marginal pool (near the vessel walls) and the circulation can occur. There are limited data concerning monocyte subtypes and septic shock, but one study showed decreased nonclassical monocytes up to nine days after onset of septic shock (40), in line with our result. On the other hand, in sepsis CD16^+^ (intermediate and/or nonclassical) monocytes have been repeatedly reported to expand (13). These results support the notion that the disease severity (sepsis vs septic shock) of the critically ill patient may influence the effector function of especially the nonclassical monocyte subtype. Therefore, stratification of patients based on disease severity will be straightforward to consider when addressing the potential role of nonclassical monocytes in disease progression.

### 4.2 Neutrophils and lymphocytes from patients have reduced CD137

To the best of our knowledge this is the first study showing that CD137 is downregulated in lymphocytes and neutrophils of septic shock patients. CD137 is thought to induce beneficial co-stimulation, promoting specific T cell activation, survival, proliferation and T cell effector functions (41). Furthermore, although the role of CD137 neutrophils is less known, a study by Sang-Chul et al. showed that stimulation of CD137 enhances activities of murine neutrophils against Listeria monocytogenes (gram-positive) pathogen (42). In line with this, another research group has shown that CD137 stimulation enhances antibacterial activities of murine neutrophils infected with the gram-positive *S. aureus* but suppresses those of mice infected with the gram-negative *E. coli (*43). These findings highlight diverse host-pathogen interactions and raise the hypothesis that pharmacologic co-stimulation of the CD137/4-1BBL pathway may help reverse immune paralysis in septic shock. It should be emphasized, however, that further research is needed to test this potential therapeutic strategy.

### 4.3 HVEM but not BTLA is regulated in patients

The HVEM receptor is another possible immunomodulator that can be translated into a medical intervention in sepsis. HVEM functions as a molecular switch, because it has both immune suppressive (BTLA) and stimulating (CD160, LIGHT and lymphotoxin-α) ligands (28). We show that septic shock patients have reduced levels of circulating monocytes and neutrophils positive for HVEM. Furthermore, our data indicated that BTLA, which is involved in the HVEM/BTLA co-inhibitory pathway, is not differently regulated in patients. This is line with Spec et al. demonstrating no difference between T-cell BTLA expression in septic patients infected by Candida Albicans and in non-septic patients (44). In line with this, another study showed no significant differences in percentage of CD4^+^ and CD8^+^ T cells that express BTLA (45). On the other hand, a study by Nicholas J. et al. showed that septic patients have increased BTLA expression on CD4^+^ T-cells and is predictive of susceptibility to secondary nosocomial infections (46). The expression of HVEM has rarely been explored in septic patients. However, one study [ (47)], conducted prior to the Sepsis-3 definition, reported no difference in HVEM expression on monocytes and neutrophils, between non-septic patients and patients with sepsis.

### 4.4 A role for VISTA?

VISTA was reduced in various immune cells of our patients. Some studies characterize this molecule as a negative regulator of T-cell responses while as an activating receptor in human monocytes (27, 48–50). If VISTA has co-stimulatory properties in monocytes and co-inhibitory in T-cells, our data suggest that monocytes and T-cells from patients exhibit immunosuppression and T-cell activation, respectively. Apparently, contradictory functions for VISTA have been reported (27). Therefore, it would be of interest to determine if VISTA has a predominantly stimulatory or inhibitory role in the pathophysiology of the septic response. Furthermore, as previously reported, myeloid cells have the highest level of expression of this protein, highlighting the function of VISTA in myeloid cell-mediated effector function. These findings warrant further investigation to clarify the functional significance of VISTA in myeloid immunity.

### 4.5 Upregulation of monocytic surface thrombomodulin

Since only patients with elevated sTM were included in this study, we further explored the surface TM. To our knowledge, this is the first evidence that shows that monocytic TM is upregulated in patients with septic shock and endotheliopathy. In fact, all subtypes of monocytes from patients upregulate surface TM, indicating a potential role of monocytic TM in septic shock induced endotheliopathy. The damage of endothelial cells and subsequent release of TM (sTM) in the study cohort, is further supported by the lack of secretion of TM by immune cells after 18 hours of *ex vivo* incubation. These findings together with the role of monocytes in the coagulation system may suggest that monocytic TM is a physiological defense mechanism against the procoagulant state in septic shock induced endotheliopathy. This is also in agreement with a study that showed decreased TM mRNA expression in peripheral monocytes correlated significantly with the severity of DIC (22). Furthermore, in patients with myelodysplastic syndromes, the presence of TM on monocytes correlated with lower-risk disease and better overall survival (51). Future studies should aim to investigate the contribution and function of monocytic TM in endotheliopathy, coagulopathy, and septic shock. One approach could be to perform *ex vivo* functional assays to explore the causal role and underlying mechanisms of upregulated monocytic TM. It should also be noted that, while sTM was selected as a representative and well-validated marker of endothelial injury, the inclusion of additional markers such as syndecan-1, angiopoietin-2, or VE-cadherin could further enhance the endothelial characterization of patients.

### 4.6 TREM-1 expression is differentially regulated on human neutrophils and monocytes

Under resting conditions, TREM-1 is expressed at higher levels in CD14^+^ monocytes compared to neutrophils, consistent with previous findings (52). Interestingly, we observed that the percentage of TREM-1 on nonclassical monocytes was significantly higher than in cells from healthy volunteers. In addition, a significant reduction of TREM-1 was observed in neutrophils from patients. Multiple factors can explain this difference in TREM-1 expression on neutrophils and non-classical monocytes. Considering the involvement of TREM-1 in effector functions and in up-regulation of the costimulatory molecules (53), the upregulation of TREM-1 on nonclassical monocytes can be important for activation and/or effector function of these cells. Regarding neutrophils, the observed decrease of TREM-1 on neutrophils from septic patients, can be due to the increased infiltration of TREM-1^high^ neutrophils, while immature neutrophils released from bone marrow (with low expression of TREM-1), are more frequent in the circulation. In support of this, Axel Bouchon et al. ( (54)) showed that neutrophils isolated from the peritoneal cavity of patients with septic shock had increased TREM-1 surface expression compared to circulating neutrophils.

### 4.7 Patients demonstrate severely impaired monocytic antigen-presenting capacity

MHC class II molecules are responsible for antigen presentation to T cells. For this reason, the evaluation of MHC class II molecules is considered the gold standard for the identification of immune dysregulation (immunosuppression and/or immunoparalysis) in patients with sepsis (16). Thus, the observed decrease of three different HLA class II clusters (DR, DQ, and DP antigens) by all monocyte subtypes from patients, underlines impaired monocytic antigen-presenting function. This aligns well with post-mortem studies of patients who died following sepsis, which shows diminished expression of HLA-DR on APCs (the monocytes being part of them) and macrophages from spleen (55).

### 4.8 The classical and intermediate subtypes were the most closely related subtypes in both healthy and septic shock patients

Based on PCA we show that classical and intermediate monocytes were the most closely related subsets. Nonetheless, the relationship between these monocyte subtypes is a subject of debate with a lack of consensus on whether the intermediate monocytes most closely resemble the classical or nonclassical (13). Differences in gating strategies and novel subpopulations within the three monocyte subtypes that cannot be identified using CD14 and CD16 alone can explain the discrepancies in the relationship between monocyte subtypes.

### 4.9 Septic shock patients exhibit hallmarks of cytokine storm syndrome

Baseline serum levels of key anti-inflammatory cytokines (IL-10 and IL-4), and 5 pro-inflammatory cytokines (IFN-γ, IL-1β, IL-6, IL-8, and TNF-α) were measured. Our findings are consistent with previous data demonstrating, both pro-inflammatory and anti-inflammatory cytokines are elevated in patients with septic shock. This “cytokine storm” that occurs in a variety of disorders may lead to multiple organ dysfunction syndrome (MODS) and early death in a subgroup of patients with sepsis (56). To link these observations to function, IL-8 and IL-6 overexpression may be the driving force behind the observed neutrophilia and reduced MHC class II expression in patients from this study, as previously reported (57, 58). Moreover, TNF-α and IL-1β have been linked to upregulating TM on macrophages and to decrease endothelial cell expression of TM, respectively (59). The opposite implication of TNF-α and IL-1β on TM may contribute to the observed increase in surface monocyte TM in this study. Nonetheless, further research is needed to explore the role of cytokines in regulating monocytic TM.

### 4.10 Implications for therapy and clinical practice: future directions

Decades of research have shown that septic shock induces immune dysregulation, characterized by an initial hyperinflammatory response followed by profound and often prolonged immunosuppression or immunoparalysis (60, 61). Although the precise mechanisms underlying sepsis-induced immune dysregulation remain incompletely understood, interconnected pathological processes - including cytokine storms, endothelial dysfunction, and immune checkpoint dysregulation - are thought to contribute to the pathogenesis of septic shock (56, 62, 63). In the present study, various pro- and anti-inflammatory cytokines were elevated and likely contribute to vascular permeability and endothelial dysfunction. Furthermore, abnormalities in TM and TREM-1 expression in some immune cells may indicate a failure of anti-coagulant and anti-inflammatory responses (64, 65), contributing to microvascular thrombosis, impaired tissue perfusion, and sustained organ failure—hallmarks of endothelial dysfunction in septic shock. These findings, together with alterations in markers (MHC II isotypes, CD137, VISTA, HVEM and BTLA) and cell populations (T cells and monocytes) associated with immune suppression, may contribute to a state of immunoparalysis. Although the pattern appears mixed and the precise mechanism behind our findings was not investigated in this study, the overall effect suggests a disruption of the regulatory signals governing T-cell and monocyte function. Such disruption may alter immune balance toward immune exhaustion, dysregulation, and ultimately immunoparalysis.

In the state of immunoparalysis, the host is unable to clear the initial infection or mount a response to secondary infections, resulting in persistent organ dysfunction and high mortality (66). Given the advances in cancer immunotherapy and the parallels between immune defects in sepsis and cancer (67), priority should be given to therapeutic trials aimed at boosting host immunity to reverse immunoparalysis, alongside pharmacological interventions to mitigate the initial hyperinflammatory response that contributes to this state. In fact, modulation of these pathways has been explored therapeutically in septic shock, for example by targeting TREM-1. Administration of high-dose Nangibotide, an antagonistic TREM-1 peptide, resulted in clinically meaningful improvements in SOFA scores among patients with higher cutoff concentrations of sTREM-1 (68). These findings have led to the need for a Phase 3 registration trial of Nangibotide in septic shock. This study, together with the observed immunologic alterations observed in the present study, underscores the need for biomarker-guided, personalized treatment. Assessing patients’ immune status using flow cytometry and functional assays can identify those with severe immune dysfunction who may benefit from targeted immune-enhancing therapies. As the immunopathogenesis of sepsis is a difficult area to investigate due to patient heterogeneity and a complex pathophysiology (69). It is now clear that one-size-fits-all therapeutic approaches, does not fit all in sepsis. Therefore biomarker-based stratification of patients will be straightforward to consider when addressing the immunological dysfunction in sepsis. Clinical outcome may be improved using a more personalized approach. Moreover, correlating these immune markers with clinical parameters such as SOFA scores or survival status could further validate their role as indicators of disease severity and prognostic predictors in septic shock. There has been growing and extensive research interest in prognostic and surrogate biomarkers, as well as in establishing correlations between companion diagnostics, clinical biomarkers, and therapeutic response. Going forward, future clinical trials in this area should expand toward dynamic monitoring, emphasizing the importance of tracking biomarker changes over time rather than relying solely on single measurements to enhance therapeutic guidance and prediction.

Addressing immunosuppression, targeting the PD-1/PD-L1 pathway inhibition appear to be well tolerated and safe for patients with sepsis or septic shock (70, 71). However, the therapeutic potential remains an area for future investigation. Our results further suggest that CD137 could be another immunotherapeutic target in septic shock. Although CD137 agonists have not yet been tested clinically in sepsis, the emergence of more well tolerated bispecific antibodies targeting CD137 in oncology offers new hope for translating this approach to the treatment of patients with septic shock (72, 73).

### 4.11 Strengths and limitations of this study

We acknowledge that this study has both strengths and limitations. The most important limitation is that the study population only encompasses a limited number of patients and that only males were included. Furthermore, only septic shock patients with severe endotheliopathy were included and, consequently, the interpretation of the results are confined to this population. It remains uncertain whether the observed immune alterations are specifically associated with endotheliopathy or represent more general immune changes related to septic shock. Incorporating a comparator group of septic shock patients with low sTM (i.e., without endotheliopathy) would provide important context for interpreting the findings. The potential impact of variations in the causes of septic shock on immunopathology has not been evaluated in this study. It is conceivable that heterogeneity in the etiology of septic shock influenced the immune profiles observed, as different pathogens or injury mechanisms may trigger distinct immune pathways and cellular responses. However, because only patients with elevated sTM levels were included, this selection may have reduced some of the variability associated with differing underlying causes. Nevertheless, further investigations with larger, pathogen-stratified cohorts are warranted to elucidate pathogen-specific immune response among patients with septic shock.

Treatments such as antibiotics or corticosteroids can rapidly influence immune phenotypes and cytokine profiles, but information on therapy before blood sampling was not recorded in this study. The study is strengthened by the comprehensive list of analyses performed, providing extensive immune profiling of well-defined patients with septic shock and endotheliopathy. Another strength is that neither selection bias nor inter-user variability was introduced in this study.

## 5 Conclusion

In this exploratory study, we observed marked changes in immune cell composition and phenotype of septic shock patients with endotheliopathy as compared to healthy volunteers. Overall, our research demonstrates that in analogy with the PD-L1-PD-1 axis, other immuno-modulators (HVEM, VISTA and CD137) prominent on myeloid cells may constitute a novel immunoregulatory system that potentially can be therapeutically explored in septic shock. However, the role and therapeutic potential of these immunological alterations provided in the present study need to be validated in a larger cohort.

## Data Availability

The raw data supporting the conclusions of this article will be made available by the authors, without undue reservation.
