# Supplementary material for: Immunophenotyping of septic shock patients with endotheliopathy: focus on monocyte subtypes and Immune regulatory molecules
Source: Front Immunol. 2025 Dec 4;16:1656815. doi: 10.3389/fimmu.2025.1656815 (PMC12711509; doi:10.3389/fimmu.2025.1656815)
Supplement: Supplementary file 1 [file DataSheet1.docx]

Supplementary Material

Immunophenotyping of Septic Shock Patients with Endotheliopathy: Focus on Monocyte Subtypes and Immune regulatory molecules

**Supplementary Table 1:**

**Panel design and Master Mix (MM) of the “TREM panel”.** Samples where stained with the indicated extracellular fluorochrome conjugated monoclonal antibodies. Besides the listed antibodies different buffers where added to the Master Mix cocktail, which were prepared right before the staining procedure.

| Target | Fluorochrome | Clone | Cat. no. |
| --- | --- | --- | --- |
| CD14 | VioBlue | REA599 | 130-110-524 |
| CD45 | VioGreen | REA747 | 130-110-638 |
| CD141 | VioBright B515 | REA674 | 130-113-666 |
| CD56 | PE | REA196 | 130-113-312 |
| CD16 | PE | REA423 | 130-113-393 |
| CD3 | PE-Vio615 | REA613 | 130-114-520 |
| CD19 | PerCP-Vio700 | REA675 | 130-113-648 |
| HLA-DR, DP, DQ | PE-Vio770 | REA332 | 130-125-984 |
| TREM | APC | REA213 | 130-101-050 |
| CD4 | VioBright R720 | REA623 | 130-127-378 |
| CD66b | APC-Vio770 | REA306 | 130-120-060 |
| Viability | 7-AAD | REA | 130-111-568 |

**Supplementary Table 2:**

**Panel design and Master Mix (MM) of the “Checkpoint panel”.** Samples where stained with the indicated extracellular fluorochrome conjugated monoclonal antibodies. Besides the listed antibodies different buffers where added to the Master Mix cocktail, which were prepared right before the staining procedure.

| Target | Fluorochrome | Clone | Cat. no. |
| --- | --- | --- | --- |
| CD14 | VioBlue | REA599 | 130-110-524 |
| CD45 | VioGreen | REA747 | 130-110-638 |
| BTLA | BV605 | J168-540 | 743986 |
| CD137 (4-1BB) | VioBright B515 | REA765 | 130-110-767 |
| CD16 | PE | REA423 | 130-113-393 |
| CD270 (HVEM) | PE-Vio770 | REA247 | 130-101-600 |
| CD66b | APC-Vio770 | REA306 | 130-120-060 |
| VISTA (PD-1H) | APC | B7H5DS8 | 2367471 |
| Viability | 7-AAD | REA | 130-111-568 |


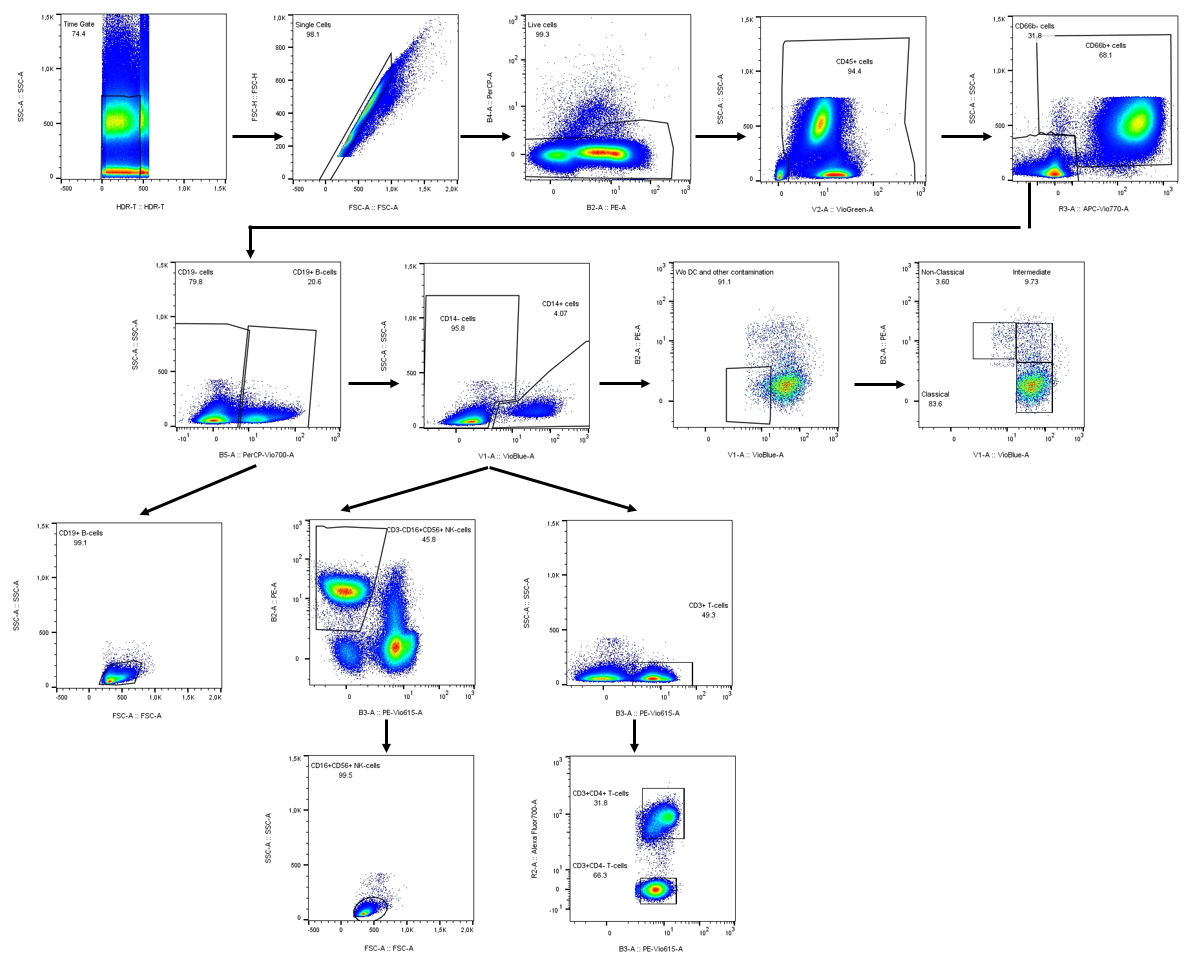


**Supplementary Figure 1. Representative flow cytometry gating strategy for the “TREM panel” to identify immune cells from whole human blood.** First bad acquired data, doublets and dead cells were excluded based on a time vs side-scatter area (SSC-A), area vs hight forward-scatter (FSC-A vs FSC-H) and 7-AAD expression, respectively. Next, platelets, debris and remaining red blood cells were excluded based on their CD45 expression. From the CD45+ leukocyte gate, neutrophils were gated based on CD66b expression. After excluding neutrophils, B-cells were identified and verified based on their expression of CD19 and by their size and granularity (FSC vs. SSC). CD19 negative cells were further used for excluding monocytes from other cells based on CD14 expression. From the CD14-negative cell population, T-cells and NK-cells cells were gated based on CD3 and CD3 vs CD16/CD56 dump channel, respectively. CD4+ and CD4- (CD8+) T cells were analyzed from CD3+ T cells using CD4 expression. Monocyte subsets were identified based on their expression of CD14 and CD16 and after having excluded potentially contaminating cells. Cells identified in this panel were then further characterized for surface markers including TREM-1, CD141 (thrombomodulin) and HLA-DR, DP, DQ, as indicated in panel design and master mix. Position of the gates was determined on the basis of fluorescence minus one (FMO) controls.


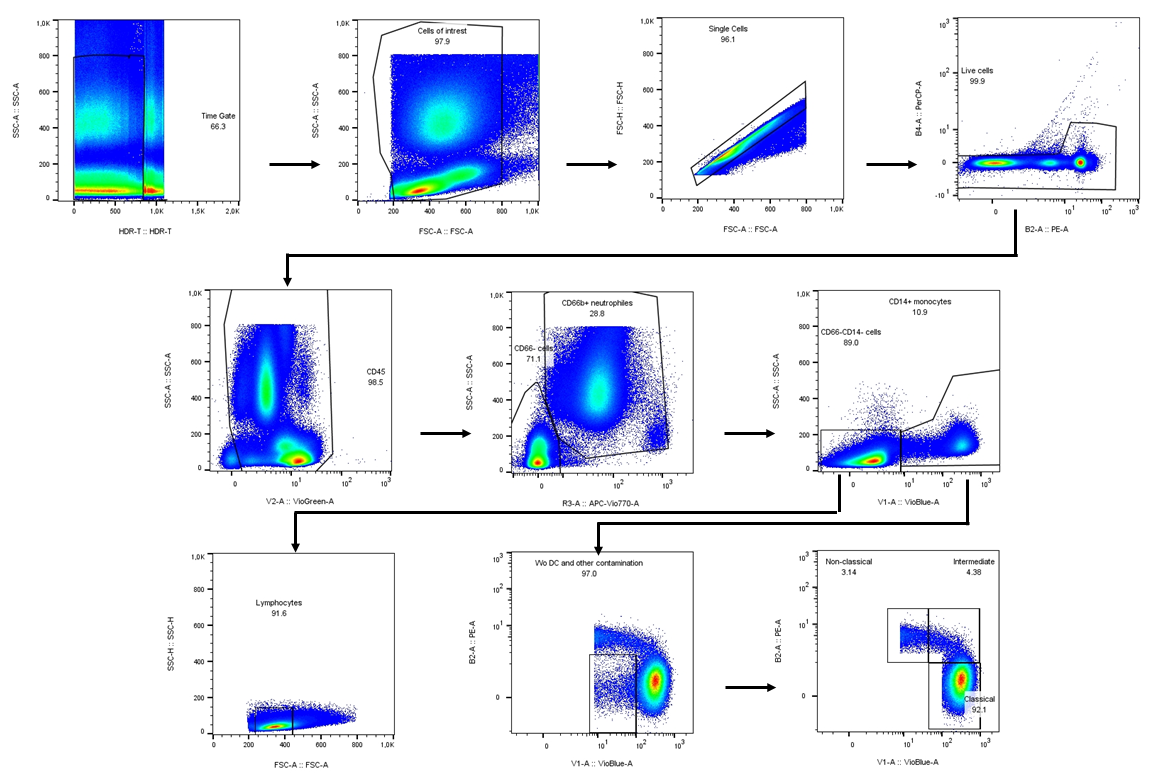


**Supplementary Figure 2. Representative flow cytometry gating strategy for the “Checkpoint panel” to identify immune cells from whole human blood.** First bad acquired data, doublets and dead cells were excluded based on a time vs side-scatter area (SSC-A), area vs hight forward-scatter (FSC-A vs FSC-H) and 7-AAD expression, respectively. Next, platelets, debris and remaining red blood cells were excluded based on their CD45 expression. From the CD45+ leukocyte gate, neutrophils were gated based on CD66b expression. After excluding neutrophils, monocytes were identified based on their expression of CD14. From the CD14-negative cell population, lymphocytes (B-cells, T-cells and NK-cells) were gated based on their size and granularity (FSC vs. SSC). Monocyte subsets were identified based on their expression of CD14 and CD16 and after having excluded potentially contaminating cells. Cells identified in this panel were then further characterized for surface markers including BTLA, CD137 (4-1BB), CD270 (HVEM) and VISTA (PD-1H), as indicated in panel design and master mix. Position of the gates was determined on the basis of respective fluorescence minus one (FMO) controls.


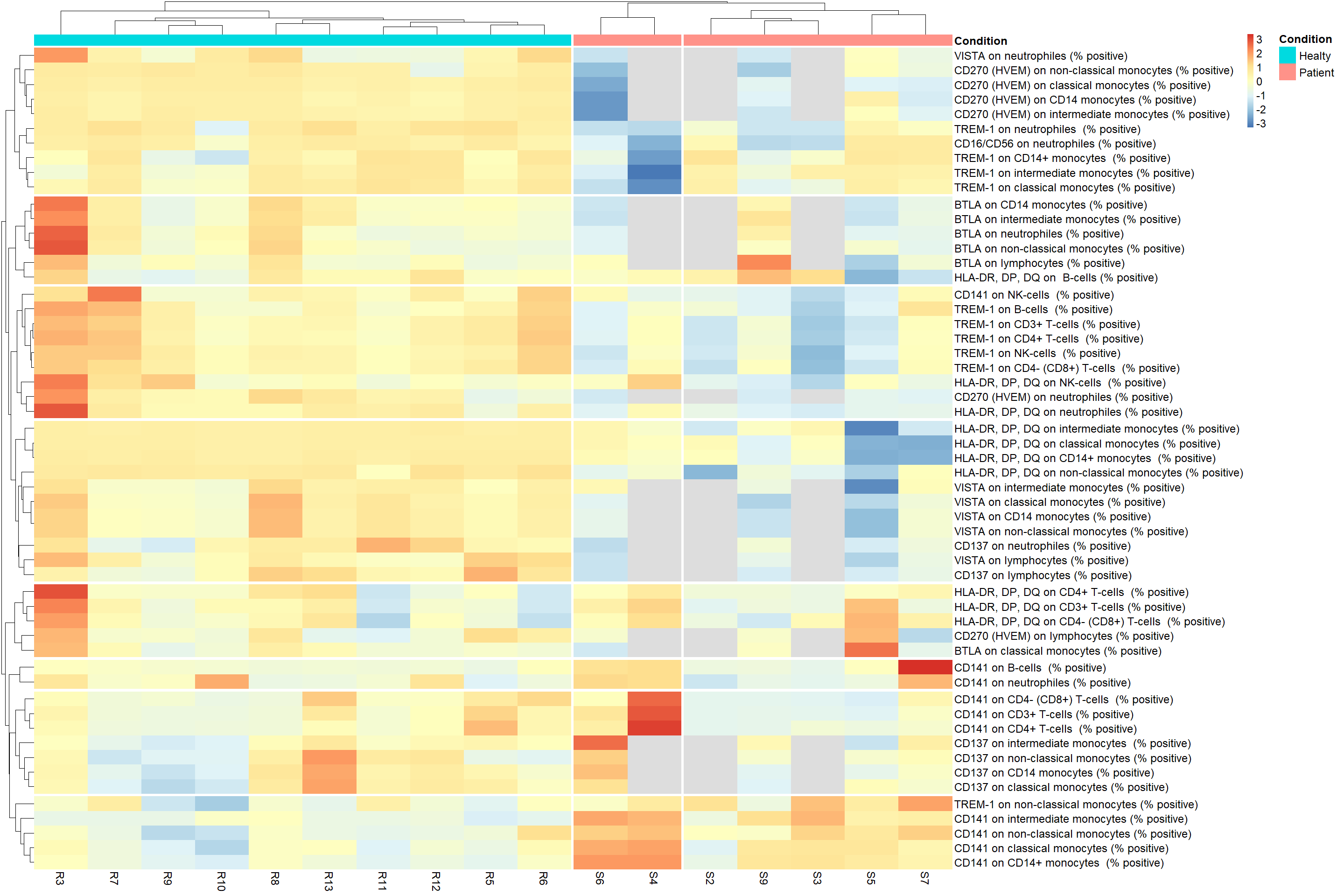


**Supplementary Figure 3.** **Unsupervised clustering of markers on immune cells can identify patients.** Heatmap of all marker used in this study on all unique cell type. In this heatmap percentage positive was used. Data was standardized. The color-scale is bounded at ± 3-fold change, with an increased percentage positive shaded red; decreased percentage positive shaded blue; unchanged percentage positive shaded yellow. The grey colour represents missing data. Furthermore, patients and healthy controls were annotated by the color skyblue and salmon, respectively.


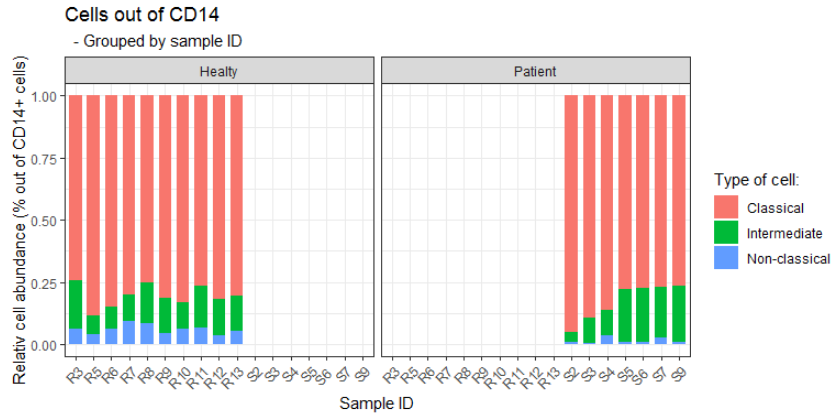

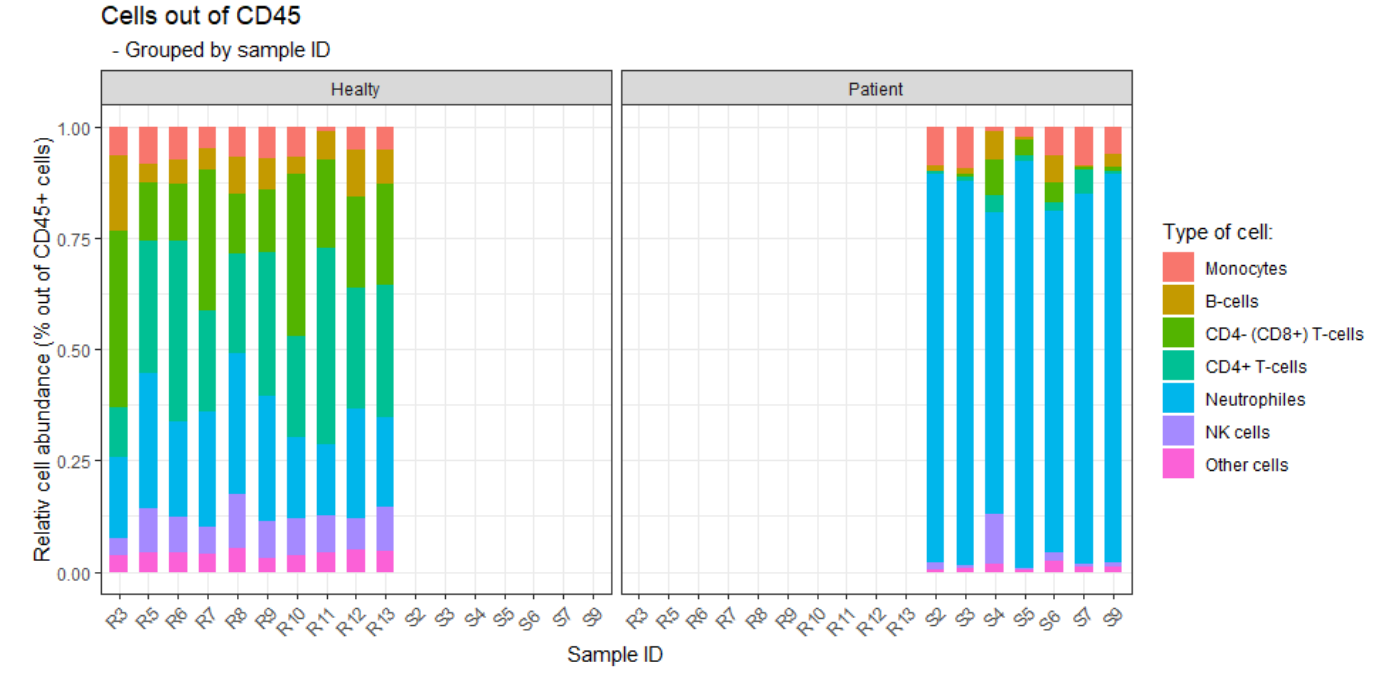


**Supplementary Figure 5.** Proportions of the monocyte subtypes in individual sample.

**Supplementary Figure 4.** Proportions of the six immune cell subsets in individual sample


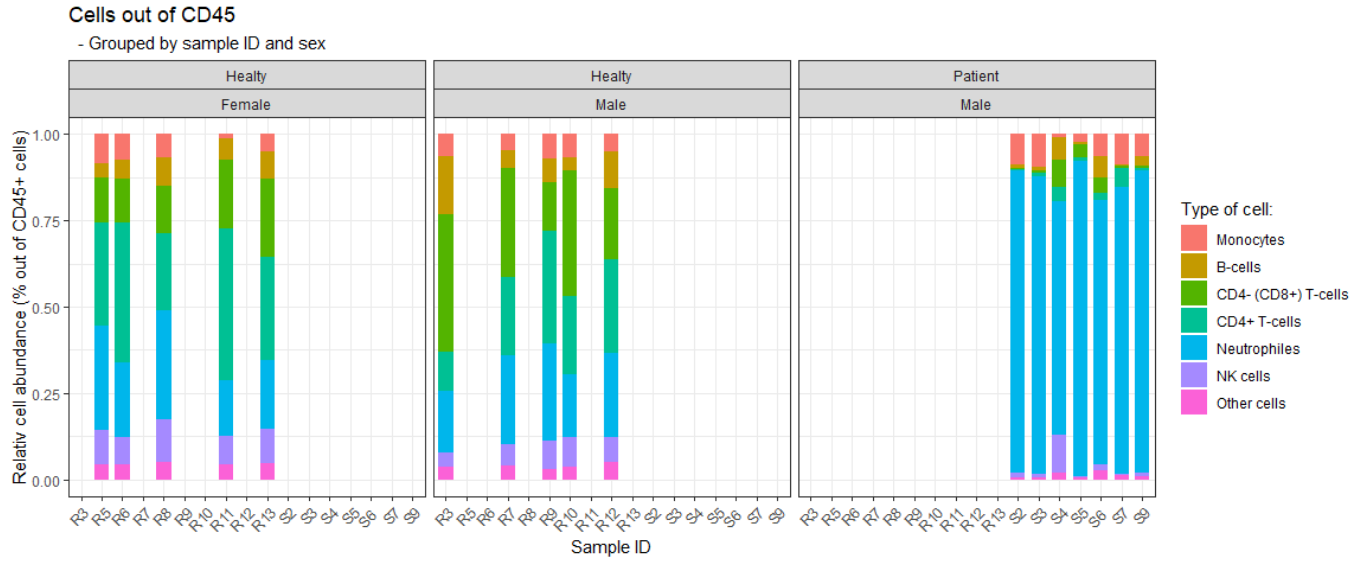


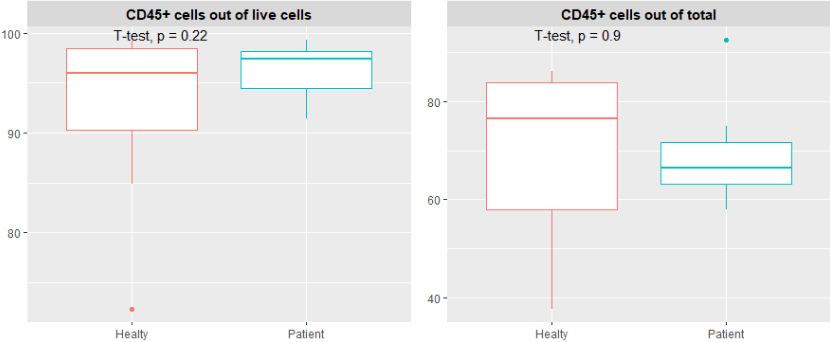


**Supplementary Figure 7.** No change in the total pool of CD45+ cells of healthy controls (n=10) and septic shock patients (n=7).


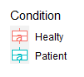


**Supplementary Figure 6. Proportions of six immune cell subsets in healthy controls (n=10) and septic shock patients (n=7) grouped by sex.** As shown previously (Klein, S., Flanagan, K. Sex differences in immune responses. Nat Rev Immunol 16, 626–638), healthy females have higher CD4+ T cell proportion than age-matched males; whereas males have higher CD8+ T cell frequencies. Proportions are defined as frequency of immune cells relatively to total CD45+ cells. Statistical significance was assessed using a Welch's t test to compare each cell type from patients with the same cell type from healthy controls (ns: p > 0.05, *: p ≤ 0.05, **: p ≤ 0.01, ***: p ≤ 0.001, ****: p ≤ 0.0001).


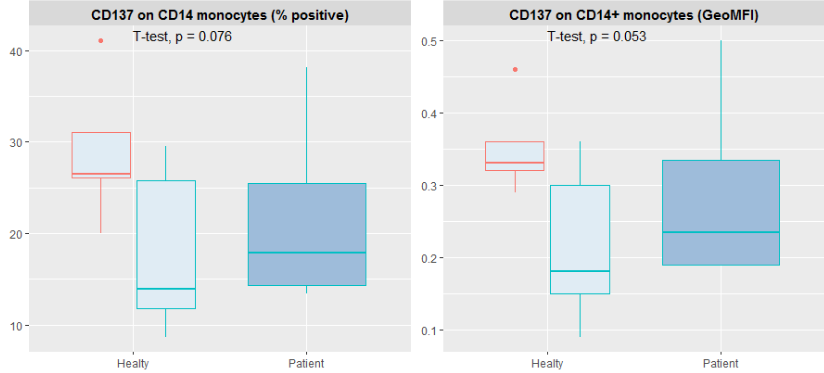

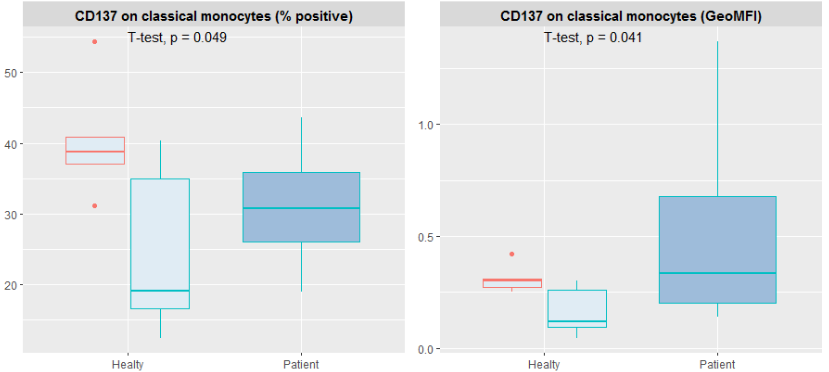

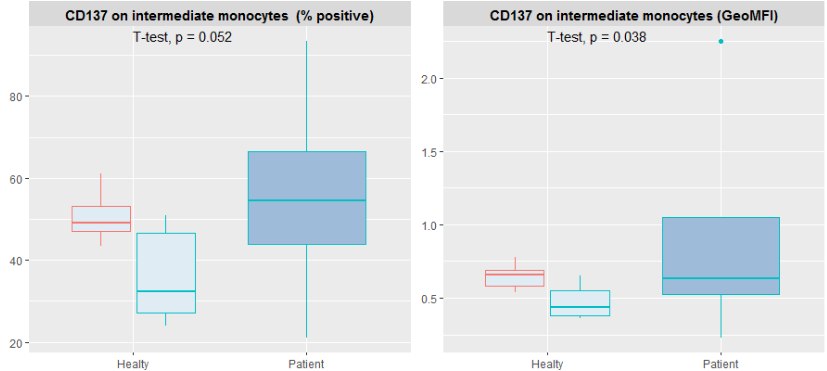

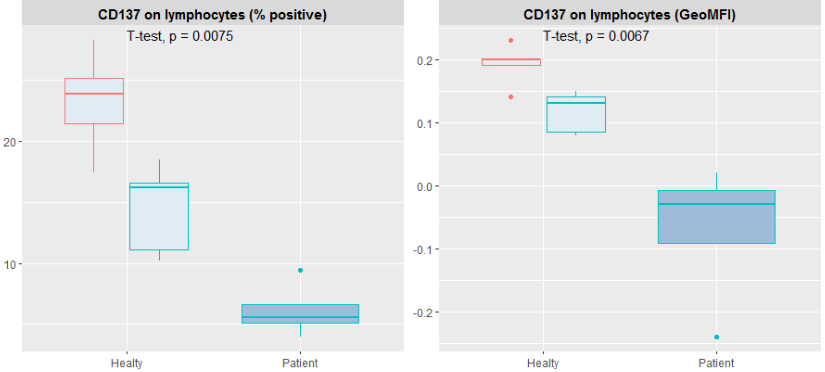


**Supplementary Figure 9. Sex immune dimorphism in costimulatory molecules and immune checkpoints in female (n=5) and male (n=5) healthy controls**. Healthy females have higher CD137 positive immune cells compared to age-matched males. Statistical significance was assessed using a Welch's t test to compare between healthy female and male (ns: p > 0.05, *: p ≤ 0.05, **: p ≤ 0.01, ***: p ≤ 0.001, ****: p ≤ 0.0001).


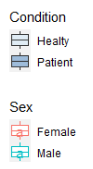

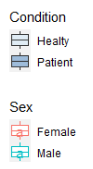

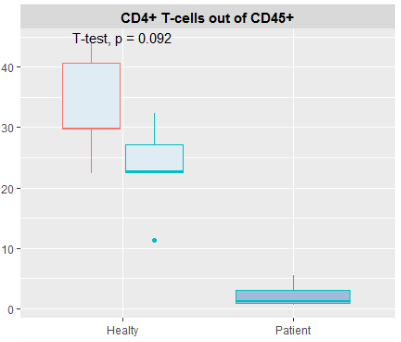

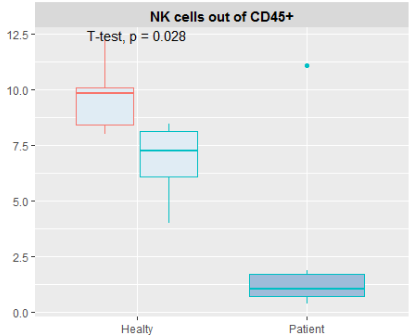

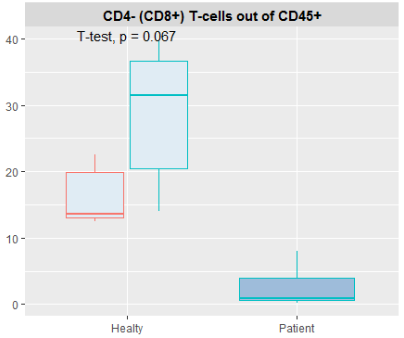


**Supplementary Figure 8. Absolute numbers of some immune cells in patients (n=7) and healthy females (n=5) and males (n=5).** Healthy females have higher CD4+ T cell number than age-matched males; whereas males have higher CD8+ T cell percentages. Absolute numbers are defined as percentage of immune cell relatively to total CD45+ cells. Statistical significance was assessed using a Welch's t test to compare between healthy female and male (ns: p > 0.05, *: p ≤ 0.05, **: p ≤ 0.01, ***: p ≤ 0.001, ****: p ≤ 0.0001).

**Supplementary Table 3.** Thrombomodulin secretion from immune cells during 18 hours incubation (37°C, 5% CO_2_) of whole blood. Not a number (NaN) were replaced by LLOD. OD_450 nm_ = optical density at 450 nm. Measurements of sTM was performed in similar manner as described in method, however this assay was performed on culture supernatant and not EDTA-plasma.

|  | Sample | ELISA reader data  (OD_450 nm_) | Soluble Thrombomodulin (ng/ml) |
| --- | --- | --- | --- |
| Control | Blank | 0.0270 | <0.625 |
| Healthy | R3 | 0.0222 | <0.625 |
|  | R5 | 0.0213 | <0.625 |
|  | R6 | 0.0194 | <0.625 |
|  | R8 | 0.0218 | <0.625 |
|  | R9 | 0.0200 | <0.625 |
|  | R10 | 0.0219 | <0.625 |
| Patient | S2 | 0.0338 | <0.625 |
|  | S3 | 0.0297 | <0.625 |
|  | S7 | 0.0221 | <0.625 |
|  | S9 | 0.0323 | <0.625 |
